# Clinical and molecular evidences of HTLV-1 infection in inpatients diagnosed with diseases previously described as associated to this infection: A case series in Gabon, Central Africa

**DOI:** 10.1371/journal.pntd.0013075

**Published:** 2025-05-14

**Authors:** Eldridge Fedricksen Oloumbou, Léonie Ledaga Lentombo, Judicaël Obame-Nkoghe, Josaphat Iba-Ba, Jeordy Dimitri Engone Ondo, Christ Ognari Ayoumi, Moussa Yaro, Abdoulaye Diané, Philomène Kouna Ndouongo, Jean-Bruno Boguikouma, Ivan Mfouo-Tynga, Augustin Mouinga-Ondémé

**Affiliations:** 1 Unité des Infections Rétrovirales et Pathologies associées, Centre Interdisciplinaire de Recherches Médicales de Franceville (CIRMF), Franceville, Gabon; 2 Service de Médecine Interne, Centre Hospitalier Universitaire de Libreville (CHUL), Libreville, Gabon; 3 Département de Médecine Interne, Université des Sciences de la Santé (USS), Libreville, Gabon; 4 Département de biologie, Université des Sciences et Techniques de Masuku (USTM), Franceville, Gabon; 5 Unité Écologie des Systèmes Vectoriels (ESV), Centre Interdisciplinaire de Recherches Médicales de Franceville (CIRMF), Franceville, Gabon; 6 Service de Neurologie, Centre Hospitalier Universitaire de Libreville (CHUL), Libreville, Gabon; George Washington University Medical Center, UNITED STATES OF AMERICA

## Abstract

The Human T-cell Leukemia Virus Type 1 (HTLV-1) infection is endemic in Gabon, and despite the high prevalence, very few cases of HTLV-1 associated diseases are sporadically described. A cross-sectional study was conducted in the main teaching hospital of Gabon. Using serological and molecular techniques, plasma samples were tested and nucleic materials of all positive samples extracted from the buffy coat, then a polymerase chain reaction (PCR) was performed to detect fragments of 220 and 522-base-pairs of HTLV-1 Tax/Rex and Env genes, respectively. From March to September 2022, a total of 352 participants (51 median age, IQR 36–62) were recruited, consisting of 290 (82.4%) patients and 65 (17.6%) patients’ family members. Of them, 36 (10.2%) samples were ELISA seropositive, and according to WB criteria, 22 were HTLV-1 positive (6.3%), 7 indeterminate (2%), 5 seronegative (1.4%) and 2 had HTLV seroreactivity (0.6%). The HTLV-1 infection was confirmed in 26 individuals (22 patients and 4 among their family members) with an overall prevalence estimated at 7.39% (26/352), and 3.7% (10/272) prevalence for diseases associated to HTLV-1, obtained from all clinical diagnoses. The link assessment between HTLV-1 infection and diseases’ occurrence revealed 10.5% of cases of Adult T-cell Leukemia/Lymphoma (2/19), 55.6% of tropical spastic paraparesis/HTLV-1-associated myelopathy (5/9) and 60% of inflammatory myopathies (3/5). Most of the detected genotypes of HTLV-1 strains belonged to the central African HTLV-1b, four defined as HTLV-1a including a-Na, a-Wa and a-TC subclades, and one belonging to the HTLV-1d. Then, assessment of HTLV-1 intrafamilial transmission and risk factors reported one case of mother-to-child HTLV-1 transmission, and significant impacts of association between HTLV-1 infection with the gender and birthplaces were observed. Here, we discussed both the prevalence of HTLV-1 infection among patients and diseases previously described as associated to this retrovirus in Gabon. Our findings highlight the necessity to develop strategies to prevent and properly manage this infection.

## Author summary

The Human T lymphotropic virus type 1 (HTLV-1) is responsible for a severe hematological malignancy and a chronic low limb disabling disease. The HTLV-1 transmission can occur through transfusion, prolonged infant-breastfeeding, and during unprotected sexual intercourse. Around 5–10 million people are infected worldwide, and the sub-Saharan region of Africa is one of the most endemic areas for HTLV-1 Infection. In Gabon, about 7.3 to 8.7% of adult populations are infected and HTLV-1 associated diseases are not properly reported; probably lesser than the reality. Thus, applying serological and molecular tools as well as assessing clinical and biological outcomes, we aimed to investigate facts and prevalences of HTLV-1 associated diseases among patients, frequently attending the main teaching hospital of Gabon. We report 7.39% of overall HTLV-1 prevalence (26/352) and 3.7% of HTLV-1 associated diseases (10/272) observed in this hospital. Equally, a great concern arose related to HTLV-1 diagnosis, with the observance of 5/8 HTLV-1 associated diseases, and those diseases remain underdiagnosed. Both, the birthplace and being a female were identified as determinant risk-factors associated with HTLV-1 infection. Our findings point out the need of strategies to prevent and effectively manage this infection in Gabon.

## Introduction

The Human T-cell Leukemia Virus type 1 (HTLV-1) is the first human oncogenic retrovirus that was discovered and isolated for the first time by *Poiesz et al.* in 1980 in the USA [1]. Globally, about 5–10 million of people would be infected by HTLV-1, and nearly half of those could be from African regions, particularly those of Western and Central Africa. In addition, HTLV-1 infection is also highly endemic in southwestern Japan, Iran, Australo-Melanesia, Caribbean basin and South American regions [2]. This retrovirus is known to be mainly transmitted through unprotected sexual intercourse (particularly from men to women), prolonged infant-breastfeeding, and contaminated blood products during transfusion, organs transplantation or intravenous drug administrations [3–7]. Although most HTLV-1 infected people remain asymptomatic throughout their lives, about 3–7% of them develop two severe diseases. As a result, a very severe hematological condition from a mature T-cell lymphoproliferation with a poor prognostic named Adult T-Cell Leukemia Lymphoma (ATL) may be manifested from 30 to 79 years of age [1,8]. While the second disease is a rare and progressive disabling neuro-myelopathy known as the Tropical Spastic Paraparesis/HTLV-1 Associated Myelopathy (TSP/HAM) occurring in 0.25% of cases [9,10]. Many other diseases like myopathies (dermatomyositis and polymyositis), arthrosis, vasculitis are also associated to HTLV-1 infection but with lesser incidences than the main two mentioned [11,12]. Some countries such as Japan, which has reported about 700–1000 ATL cases reported per year has a people living with HTLV-1 estimated to be more than 1.08 million. Surprisingly, the African continent is supposed to house the most people living with HTLV-1 worldwide, has fewer ATL and TSP/HAM cases reported until now [13–15].

Located in Western Equatorial Africa, Gabon is a country that has an estimated population of over 2 million inhabitants and a density of 8,2 habitants per Km^2^, leaving about 80% of land covered by the tropical forest [16]. Although the relatively low population, Gabon is undoubtedly one of the highest endemic area for HTLV-1 infection in the world with prevalence rates in the general adult population ranging from 7.3 to 8.7% [6,17]. Despite these high prevalences, no health policy is available to reduce HTLV-1 transmission in the country. Moreover, no systematic screening of HTLV-1 is implemented in sanitary facilities such as for blood donors and pregnant women in hospitals [7,18]. Thus, the HTLV-1 pathogen remains until now, an unknown virus by the Gabonese community; the general population and most of local physicians [18]. Therefore, one of the direct consequences of this limited awareness could be an under-estimation of cases of HTLV-1 infection and associated diseases in this endemic country. Previously, Cassar and Gessain estimated the number of HTLV-1 infected people in Gabon between 16,000–30,000 in 2012 [2]. Thus, considering the occurrence of ATL and TSP/HAM among carriers, the numbers that would have been reported until now are estimated to be 1/1000 and 1/30,000 per year; at least 16–30 ATL and 1 TSP/HAM cases, respectively. Since 1986, date of the first study on HTLV-1 infection in Gabon until nowadays, only 13 cases of HTLV-1 related diseases have been reported in this endemic country, consisting of 5 TSP/HAM and 8 ATL [19–23]. Furthermore, most of these cases were reported during epidemiological studies carried out on HTLV-1 infection. So far, a few studies combining both clinical and epidemiological approaches have been conducted to investigate the prevalence of HTLV-1 infection among people living with diseases, previously described as associated to this retrovirus, in one hand, and there is no study that highlights the situation of underdiagnosis of this infection in sanitary facilities of Gabon, in the other hand.

Thus, we performed a clinical and epidemiological investigation among inpatients and patients regularly treated at the main teaching hospital of Gabon (Centre Hospitalier Universitaire de Libreville, CHUL). We aim to evaluate the overall prevalence of HTLV-1 infection among patients at 3 main units of the CHUL (Neurology, Internal Medicine, and Dermatology) by using both serological and molecular tools for diagnosis. Then, considering the positivity to HTLV-1 we determined the prevalence of the infection in diseases observed in these 3 units, before comparing the clinical and biological outcomes of HTLV-1 positive patients suffering from diseases that were previously HTLV-1 associated. Epidemiological analysis was finally conducted to identify the risk-factors for HTLV-1 among these patients, while molecular and phylogenetic aspects were utilized to characterize and determine HTLV-1 circulating genotypes and HTLV-1 intrafamilial transmission, respectively.

## Materials and methods

### Ethics statement

This study was approved by the National Ethics committee of Gabon, registered as PROT N°0020/2020/PR/SG/CNER. Prior to getting written consent forms and blood samples, the aim of this study was clearly explained to each participant. Similarly, written informed consents for children and unconscious patients were obtained from their parents, recognized guardians or the person’s close-relative “next of kin”.

### Study area and population

Between March and September 2022, we conducted a cross-sectional study in the Centre Hospitalier Universitaire de Libreville (CHUL), the main teaching hospital of Gabon, localized in the capital city, Northwest littoral of the country. Participants were recruited among patients treated at the Neurology, Internal Medicine and Dermatology units of this hospital. The criteria of inclusion were: i) being treated (hospitalized or not) in one of the 3 above-mentioned units of CHUL, ii) suffering from any of the diseases described as associated with HTLV-1 and iii) be willing to participate in the study. To investigate vertical transmissions as well as the circulation of HTLV-1 among individuals from the same family, patients’ relatives and/or siblings who were present at the hospital, were encouraged and invited to participate before the confirmation of HTLV-1 diagnosis. Patients’ family members, who did not show any sign of HTLV-1 associated diseases or whatsoever were designated as “asymptomatic” in this study.

### Data collection, clinical and biological examinations

Patients were examined by physicians of their respective units, and confirmations of diagnosis were made based on medical analyses of biological results.

A standardized survey/questionnaire was used to collect personal information, epidemiological data and identify the risk-factors associated with HTLV-1 infection. A volume of 5 to 7.5 ml of blood sample was collected on ethylenediaminetetraacetic acid (EDTA) tubes from each participant. Within 12 to 24 h after sampling, all EDTA tubes were centrifuged at 2000 rpm for 15 min, then volumes of 1.5-2 ml of plasma and 1-1.5 ml of buffy coat were separated by using single-use Pasteur pipets. Next, all plasma and buffy coats were firstly frozen at -20°C at CHUL for around 2 weeks, before transfer to CIRMF facilities, where all samples were stored at -80°C until HTLV-1/2-assessment and further analysis.

### HTLV-1 serological analysis or diagnosis

The HTLV-1 diagnosis was initiated by test screening HTLV-1/2 antibodies in all plasma samples from participants, using the ELISA kit (HTLV-I/II ELISA 4.0, MP diagnostic, Ref# 23080-0961). Positive samples were further tested by Western Blot (WB) assays (HTLV BLOT 2.4, MP diagnostic, Ref# 11080-036) to confirm and differentiate between HTLV-1 and HTLV-2 infection. Obtained results from WB assays were interpreted according to the manufacturer’s instructions. Thus, plasma samples were considered HTLV-1 positive when displaying reactivity to p19 gag antigen and two env (GD21 and Rgp46-I also named MTA-1) bands with or without p24 gag antigen. Similarly, HTLV-2-positive samples were defined when displaying reactivity to gag p24 antigen and two env (GD21 and Rgp46-II) bands with or without p19 gag antigen. Samples displaying reactivity to both gag (p19 and p24) antigens and GD21-env bands were considered as HTLV positive but not able to be assigned to a particular type (untypeable). Samples with only partial reactivity to some of these viral proteins (GD21, Rgp46-I and/or Rgp46-II, and without reactivity to gag proteins), were defined as indeterminate.

### Molecular diagnosis of HTLV-1

High molecular weight DNA was extracted from peripheral blood buffy coats (PB-BC) of all ELISA-positive samples by using the QIAamp blood Mini kit (Qiagen, Courtaboeuf, France). The DNA concentration was quantified with a Nanodrop spectrophotometer (Thermo Fisher Scientific1) instrument, as described previously, and the genetic integrity was evaluated by amplifying an Albumin gene fragment [24]. Then, two successive HTLV-DNA polymerase chain reactions (Nested PCR) were performed using generic system with consensus primers (AV45/AV46 and AV42/AV43) amplifying a 220-base pairs (bp) fragment of tax/rex, according to protocols previously described [25,26]. After obtaining the generic PCR products, a semi-nested PCR was performed using Env1/ Env22 as outer primers and Env1/ Env2 as the inner primers, for amplifying a 522-bp fragment of HTLV-1 Env gene, as previously described [27,28].

### Positivity criteria for HTLV-1 infection

Examining serological and molecular results, participants were considered infected with HTLV-1 if their WB profiles were HTLV-1, either HTLV-1/2 or HTLV. Furthermore, participants with an indeterminate WB profile but associated with HTLV-1 positive PCR, were also considered HTLV-1 infected.

### Sequencing and phylogenic analysis

Only PCR products of expected sizes were sent to Macrogen Europe B.V (Meibergdreef 57, 1105 BA Amsterdam, Pays-Bas) for sequencing and back to CIRMF for phylogenic analysis. All sequences were analyzed with ChromasPro 1.7.7 software to get consensus sequences, then HTLV-1 genotypes were identified by BLAST search and to obtain sequences using the GenBank database (http://www.ncbi.nlm.nih.gov/BLAST). Then, multiple sequence alignments of 522-pb fragment of Env gene were performed with the ClustalW algorithm (v 1.6) from the Mega 7.0 software and Maximum Likelihood method was used to infer the phylogenetic topology. A bootstrap adjusted with 1,000 replications was implemented to assess the robustness of the tree topologies.

### Statistical analysis

Data were recorded in an Excel file and analyzed using R software version 4.0.2 (http://cran.r-project.org). We employed the Akaike Information Criterion (AIC) to select the best-fitting model, analyzing changes in AIC with the ‘dredge’ function from the MuMIn package [29]. This analysis identified two of the twelve recorded independent variables as significant influencers of the prevalence of HTLV-1 infection, which were then included in the final model. Models with the lowest AIC and using the fewest possible independent variables were most suitably deemed, with ΔAIC values below two and also considered relevant. To assess the significance of differences in the distribution of HTLV-1 infection in each model, we applied the Chi^2^ significance test (p < 0.05). In this work, although others may exist, only ATL (gathered with non-Hodgkin Lymphomas), TSP/HAM (included in the diagnosis of low limbs motor deficits) and inflammatory myopathies were considered as HTLV-1 associated diseases. Due to a low number of recorded cases of these diseases, a Fischer test was used to assess the association between HTLV-1 infection and diseases. However, we also used a Chi-square test (χ^2^) to assess the association between HTLV-1 and some potential risk-factors, whenever applicable. Thus, the degree of statistical significance was considered when *p-value* < 0.05. An univariable analysis was performed to evaluate the strength association between HTLV-1 positive status and risk-factors, which was estimated as adjusted odds ratio (AOR) with a confidence interval at 95% (95% CI).

## Results

### Descriptive analysis of studied population

#### Repartition of sampled individuals according to socio-demographic criteria.

Among 352 recruited individuals, there were 155 men and 197 women with a median age of 51 years (IQR 36-62). Most of them, 92.3% were Gabonese citizens (311/337), born and grown up in the country. The remaining individuals, about 7.7% were African citizens (26/337) from mainly West Africa including Mali and Senegal. Distribution per medical units, shown in Table 1 below, reports that 53.7% of patients were treated at the Neurology unit (189/352), then 43.2% at the Internal Medicine (152/352), and the final 3.1% at the Dermatology unit (11/352). Among participants, around 17.6% of collected samples were from patients’ relatives and/or siblings (62/352). We observed that 98% of patients had only one disease (260/265), while 1.5 and 0.3% were found with 2 and 4 diseases (4/265 and 1/265), respectively.

**Table 1:** Socio-demographic parameters of the studied population.

Variable	Category	N	%
**Gender**	Men	155	44
	Women	197	56
**Age**	[0-14]	6	1.7
	[15-30]	54	15.3
	[31-46]	88	25
	[47-62]	125	35.5
	≥63	79	22.4
**Manifestation**	Asymptomatic	62	17.6
	Patients/Symptomatic	290	82.4
**Nationality**	^*^Other citizens	26	7.7
	Gabonese citizens	311	92.3
**Unit**	Dermatology	11	3.1
	Internal Medicine	152	43.2
	Neurology	189	53.7
**Clinical diagnosis number per patient**	1	260	98.1
	2	4	1.5
	4	1	0.4

**N**: size

**%**: Percentage

* **Other citizens:** includes patients born in Benin, Burkina Faso, Cameroon, Congo-Brazzaville, Ivory Coast, Equatorial Guinea, France, Mali, Nigeria, Senegal, and Togo.

### Repartition of sampled individuals according to the clinical diagnosis

A great variability of diagnosis was observed among participants, despite this great variability, 25 out 290 patients had not yet received a precise diagnosis at the time of the sampling. Thus, a total number of 272 different types of clinical diagnoses was recorded for 265 patients (S1 Fig). The most prevalent diagnosis observed with 87 patients (32%) was ischemic stroke, followed by hemorrhagic stroke with 25 patients (9.2%). Already reported pathologies associated with HTLV-1 infection like low limbs motor deficit for TSP/HAM (9/272), non-Hodgkin’s lymphoma for ATL (19/272), inflammatory myopathies (5/272) or arthrosis (3/272) represented 3.3, 7, 1.8 and 1.1% of all recorded diagnosis, respectively. We also reported some vasculitis cases (3/272) which represented 1.1% of all recorded clinical diagnoses. However, it is important to note that patients with more than one diagnosis were included in the respective diagnoses.

### HTLV-1 serological and molecular results

Among the 352 plasma samples tested (ELISA), 36 samples (10.23%) were seropositive to anti-HTLV-1/2 antibodies. Whereas only 22 samples among them were HTLV-1 positive by Western blot (WB), as per manufacturer’s criteria. Also, 2 samples presented an HTLV profile, 7 were indeterminate and 5 seronegative for the same test.

Molecular tests performed on tax/rex and env regions confirmed a HTLV-1 and/or HTLV infection for 26 samples among all 36 ELISA positive. In other words, all samples with confirmed HTLV-1 [22] and HTLV [2] WB profiles, were amplified and 2 other amplifications were obtained with indeterminate profiles (Table 2). Thus, the outcome of serological test (WB) and molecular analysis by PCR on these two regions allowed to estimate an overall HTLV-1 prevalence rate of 7.39% (26/352).

**Table 2:** PCR on tax/rex and env regions results according to the WB profiles.

		PCR results		n+ (%)
**WB profiles**	profile Number	Tax/Rex	Env	
HTLV-1	22	22	15	22
HTLV	2	2	2	2
INDETERMINATE	7	2	2	2
NEGATIVE	5	0	0	0
Total	36	26	19	26/36 (72.22)

**n + : number of positive samples to HTLV-1 found**

**%**: the proportion of at least one PCR positive result (on Env gene or tax/rex region) according to WB profiles.

### HTLV-1 description prevalence according to clinical diagnosis

As previously indicated, 265 among the 290 patients received at least one diagnosis during this study. A total number of 272 disease cases were identified from all 265 patients, and 22 patients were HTLV-1 positive. For those 22 HTLV-1 infected patients, 25 different diagnoses were recorded (Table 3 below). The prevalence of diseases considered associated with HTLV-1 in this work was estimated to be 3.7% of all recorded diagnoses [10]/272) including non-Hodgkin’s lymphoma (2/19), low limbs motor deficits (5/9), and inflammatory myopathies (3/5). Table 3 below presents different clinical diagnoses and prevalence rates recorded among the 22 patients infected with HTLV-1.

**Table 3:** Clinical diagnoses and prevalence rates recorded in the 22 HTLV-1 positive patients.

	Number (N)	n+ (%)	(95% CI)	P-value
**Clinical diagnosis**			-	
Autoimmune hemolytic anemia	11	1 (9.1)		
Arthroses	3	1 (33.3)		
Hemorrhagic stroke	25	1 (4)		0.07
Ischemic stroke	87	3 (3.4)		
Sickle cell disease	5	1 (20)		
Myeloide leukemia	13	1 (7.7)		
Lupus	15	2 (13.3)		
Non-Hodgkin’s lymphoma	**19**	**2 (10.5)**	(1.3-33.3)	
Inflammatory myopathies	**5**	**3 (60)**	-	
Parkinson	4	1 (25)		
Immun thrombocytopenic purpura	5	1 (20)		
Kaposi’s sarcoma ± other	2	1 (50)		
Low limbs motor deficit	**9**	**5 (55.6)**	(21.2-86.3)	
Brain tumor probable	2	1 (50)	-	
Vasculitis	3	1 (33.3)		
Total	272	25		

**N: total number of recorded diseases, n + : number of diseases with HTLV-1 positivity**

### Clinical characteristics of HTLV-1 positive patients with low limbs motor deficit

Among the 9 patients with low limbs motor deficit, 5 were HTLV-1 positive and considered to be suffering from probable TSP/HAM according to the criteria described by Castro-Costa and co-workers [30] (Table 4). Here, it was about 5 Gabonese women with mean 40.7 ± 10.1 of age on disease onset (for 4/5) and mean disease evolution time of 11.7 ± 9.1 years. All these positive patients were originated from different provinces of Gabon, naming Estuaire, Haut-Ogooué, Ogooué-Maritime, Moyen-Ogooué and Woleu-Ntem. Two of these HTLV-1 positive patients (2/5, N°277 and N°259) (S2A–S2C Fig) reported a gradual onset of pain and symmetric low limbs weakness, which became disabling after 2–3 months of evolution. However, two other patients (N°218; S2D Fig, and N° 233) reported feeling an electric shock from right iliac fossa, on the onset disease before progressive sensation of pains and low limbs weakness leading to the disabling some months later. The most insidious onset disease was that of patient (N°19), who started having severe headaches followed by a total visual blur to the left eye at 34 years old. Nearly 10 years after those first symptoms, the disabling condition has progressively started to give way to generalized weakness and low limbs oedema, appearing during walking in most cases.

**Table 4:** Clinical and biological characteristics of probable TSP/HAM patients.

	Sample numbers				
	N°19	N°218	N°233	N°259	N°277
**Characteristics/Criteria**					
Age (years)	44	51	82	67	48
Gender	Woman	Woman	Woman	Woman	Woman
Reason of consultation	Alteration of the general condition in known patient and follow-up for polymyositis	Follow-up of polymyositis	Right low limb motor and sensory deficits	Low limbs motor deficits	Low limbs motor and sensitive deficit
Disease evolution time	10 years	22 years	–	2 months	15 years
Presence of Babinski sign	Yes	–	No responsiveness	Yes	Yes
HTLV-1 antibodies in cerebrospinal fluid (CSF)	–	Yes	–	–	–
HTLV-1 antibodies in plasma and positive PCR	Yes	Yes	Yes	Yes	Yes
Insidious onset	Yes	No	No	Yes	Yes
Bladder disturbances: urinary incontinence and/or retention	Yes	Yes	–	Yes	Yes
Sphincter disturbances: constipation or fecal incontinence	Yes	Yes	–	Yes	Yes
Low back pain	Yes	Yes	Yes	–	Yes
Proximal weakness in the lower limbs	Yes	Yes	–	Yes	Yes
Hyperreflexia of the lower limbs ± clonus	Yes	Yes	Yes	Yes	Yes
Hyperreflexia of the upper limbs	No	Yes	–	–	Yes
Sensory disturbances	No	Yes	–	–	Yes
^*^MRI results (thoracic & lumbar)	–	without particularity	–	–	without particularity
Cognitive impairment	No	No	–	Yes	Yes
Dementia	No	No	–	Yes	No
Muscular atrophy	Yes	No	–	No	Yes
Other associated clinical signs	Polymyositis	Polymyositis	–	No	–
Other systemic manifestation	Probable uveitis	Sjörgen syndrome	–	No	–
Assessing to walking	Daily in wheelchair	Walking with bilateral supports	Daily in wheelchair	Daily on a bed	Daily in wheelchair
HIV serology	Negative	Negative	–	–	Positive
Pott’s disease	Negative	Negative	–	–	Negative
Intra family antecedent of spastic paraparesis	No	No	–	No	Probably (an uncle)
Alive/Dead	Alive	Alive	Alive	Dead	Alive
**Conclusion**	Probable TSP/HAM	TSP/HAM	Probable TSP/HAM	Probable TSP/HAM	Probable TSP/HAM

* MRI: Magnetic resonance imaging, HIV: Human immunodeficiency virus.

Chronic constipations were noticed in 3/4 of patients, while fecal incontinence in 1/4 of them. However, both urinary incontinence and retention were noticed in 2/4 of patients, while only retention was reported for the other two patients (2/4) (Table 4). Excepted for 1 patient (N°277), all remaining HTLV-1 positive patients (4/5) were HIV-1/2 seronegative. The Table 4 below summarizes clinical and biological outcomes of these 5 HTLV-1 positive patients suffering of low limbs motor deficits.

### Clinical characteristics of HTLV-1 positive patients with non-Hodgkin Lymphoma

We recorded 19 non-Hodgkin’s lymphoma during our sampling time and two of them were positive to HTLV-1 infection. Clinical and biological characteristics of these two HTLV-1 positive patients were used according to 1 point-score classification from Levine and co-workers [31], and the classification outlined by Shimoyama and co-workers [32], led to a probable ATL diagnosis and investigation of clinical ATL subtype forms (Table 5).

**Table 5:** Diagnosis of ATL subtype among HTLV-1 positive patients with non-Hodgkin lymphoma.

	Patient Number	
	N°142	N°148
**Hematologic parameters**		
Age (years)	48	40
Evolution time	9 years	3 months
HTLV-1 positive PCR	Yes	Yes
Lymphocytes’ concentration	2256,94/µl	15236/µl
Abnormal T-lymphocytes (smear blood)	Some large hyper-basophilic T cell	27.5%
Flower cells	No	Yes
LDH value (UI/L)	Normal (397)	–
Calcemia (mmol/L)	Normal (2.25)	Normal (2.2)
**Others involvement**		
Skin lesions	No	No
Lung lesions	No	–
Lymph node	Yes	Yes
Hepatomegaly	No	Yes
Splenomegaly	No	Yes
Central nervous system	No	No
Bone	No	–
Ascites	No	Yes
Pleural effusion	No	Yes
Gastrointestinal	No	Yes
Alive/Dead	Alive	Dead
**Conclusion on ATL subtypes**	**Lymphoma form**	**Acute form**

LDH: lactate dehydrogenase

The two patients were both Gabonese men aged 48 and 40 years-old (N°142 and N°148) from the southeast and the northern regions of the country (Ogooué-Lolo and Woleu-Ntem provinces). One of these two patients (N°148) presented a typical and acute ATL form, the first symptoms of the disease began in February 2022, around 3 months before his hospitalization at CHUL, due to movable and painless lymph nodes, which had gradually increased. Thus, by May 2022, the symptoms had worsened with a general alteration that was characterized by a weight loss as asthenia, night profuse sweats, appearance of cervical oedema with axillar, and inguinal lymph nodes. A computerized tomography scan (CT scan) of abdominal-pelvic region was realized for abdominal pain and lymphadenopathies, revealing a bilateral medium and abundance of pleural effusion as well as abdominal-pelvic ascites and micro inter-aortico-caval lymphadenopathies in mesenteric with homogenous hepatosplenomegaly. Peripheral blood analysis reported lymphocytosis to 15236/µl with 27,5% of abnormal lymphocytes with nuclear morphology like a flower (S3 Fig). After screening, the patient was seronegative for all HIV-1/2, HBV, HCV and syphilis antibodies. Histopathological analysis from two nodular fragments of cervical biopsy could not firmly confirm an ATL diagnosis but mainly revealed the proliferation of T-cell tumoral lymphocytes (medium and small with atypical nuclei). Unfortunately, the patient passed on a few moments after hospital admission and start of chemotherapy (cyclophosphamide, vincristine and prednisone).

After a suspicious of an ATL case, the second patient (N°142) was examined, a diagnostic of a probable lymphoma was established while the ATL classification was «inconsistent with ATL» due to numerous diagnostic limitations. The story of the patient started in 2013 with the appearance of cervical, bilateral, axillary and inguinal lymph nodes, mobile and painless with regular episodes of fever. It was at the end of the same year that he tested positive for HTLV-1 after ELISA test during a blood donation. The presence of lymph nodes was confirmed in 2018 and the diagnosis of a T-cell lymphoma was also established after medical examinations that led to the detection of anti-HTLV-1 antibodies. During a disease follow-up session in 2022, several symptoms were observed including unilateral eye pain with blurred vision, tear effusion and red sclera. However, the patient also complained about continuous constipation and regular lower extremity muscle pain, characterized by excruciating cramps. The HIV1/2, HCV, HBV and syphilis antibodies screenings were seronegative, while the lymphocytes’ concentration was estimated to 2256,94/µl, which was normal as well as the clinical examination and medical imaging of the liver and spleen.

### Clinical characteristics of HTLV-1 positive patients with inflammatory myopathies

During the sampling period, 5 patients suffering from inflammatory myopathies including 2 cases of dermatomyositis and 3 cases of polymyositis, were frequently monitored. Serological and molecular results showed HTLV-1 positivity only for those with polymyositis. Here, we describe the clinical characteristics of these HTLV-1-positive patients found with polymyositis.

#### Case 1 (n°19).

Originated from the coastland province of Ogooué Maritime, a 44-year-old female teacher was treated at the Internal Medicine unit for polymyositis. The pathology was said to have started in 2012 with violent headaches, followed by a total blurring of vision in the left eye. Several efforts, like MRI scanning and assessment, were done to elucidate the cause but those attempts were not conclusive. The phase of development of the pathology can be considered as the period between the appearance of the first symptom in 2012 and the worsening phase in latter 2021. That period was mainly marked by a regular appearance of a generalized muscular weaknesses with edema on the lower limbs, mostly appearing when walking. The diagnosis of polymyositis was made in 2014 and based on the biopsy of the deltoid muscle, which showed inflammatory infiltrations as well as positivity for antinuclear antibodies. From 2021, the patient started to complain about regular cramps affecting both lower limbs. Then, a sudden appearance of high intensity arthralgia associated with myalgia (pain rating 7/10) and muscular weakness occurred, forcing the patient to remain in hospital in 2022. Despite the administration of therapies based on corticosteroids, chemotherapies and immunosuppressant (methylprednisolone, cyclophosphamide and imurel), the clinical condition continued to deteriorate, and the patient had become unable to get started on her own with persistent weakness in the lower limbs after two weeks of hospitalization. Given the fact that the serology results were negative for anti-HIV1/2, HBV and HCV antibodies, then the clinical examination carried out by the neurologist revealed muscular strength at 2/5 in both lower limbs, damage to the pyramidal tracts that was characterized by a positive Babinski sign, coupled with lively achilean-type osteotendinous reflexes and patella, allowing the diagnosis of a spastic paraparesis to be retained.

#### Case 2 (n°100).

The second case is that of another 48-year-old teacher, originated from the province of Ogooué-Lolo, taken from the dermatology ward, where she was hospitalized for grade 4 buttock sores. She was quadriplegic and had received the diagnosis of inflammatory myopathy, whose symptoms would have started 8 years before her hospitalization. The history of the pathology would then have started at the age of 40 with cramps affecting both lower limbs, progressively and periodically, most often at night, forcing the patient to self-medicate with magnesium. The evolution of the pathology under this treatment was marked by a regression of symptoms. One year later, the patient noticed a progressive onset of muscular weakness in the lower limbs, leading to a loss of balance. This resurgence of symptoms motivated the patient to book for a medical consulting session, where an MRI was requested, and the result was not significant. Four years later, she also noticed a decrease in muscular strength in the upper limbs, prompting another consultation, where vitamins (B1, B6, and B12) were prescribed but without real effectiveness. Then, the evolution of the pathology under this treatment was marked by a persistence of symptoms and an alteration in the patient’s quality of life, thus forcing her to be hospitalized. Her clinical examination by the neurologists revealed muscular weakness in all four limbs with a strength assessment of 1/5. Hypertonia of all four limbs was also observed as well as amyotrophy of the deltoid muscle, thoracic region, interosseous muscles, quadriceps and forearms (S4A and S4B Fig). There were no sensory disturbances or damage to the pyramidal pathways. However, an elimination of the idiomuscular response was noted as well as a proximo-distal motor deficit. Serum biological examinations of the patient revealed positivity for anti-HBV antibodies and an increase of serum protein levels to 84g/L with high gamma globulin fraction. The autoimmune assessment showed positivity for anti-nuclear antibodies and the biopsy of the deltoid muscle reported a severe muscular atrophy, suggesting dystrophic myotonia or chronic neuropathy. At the same time, an ELISA test to detect anti-HTLV-1/2 antibodies was requested and returned positive. Despite the administration of antiretrovirals (zidovudine, lamivudine), interferon-alpha immunomodulators, anti-inflammatories, corticosteroids, and the patient’s condition considerably deteriorated, and she passed on two months after returning home.

#### Case 3 (n°218).

The last case of polymyositis is that of a 51-year-old female nurse from the Estuaire province, whose pathology began at the age of 29, when she felt a sensation of electric discharge in the right fossa or iliac bone during her working time, which was followed by a feeling of muscular weakness leading to a loss of balance and the patient’s downfall. This phase of initiation of symptoms was followed by a long period (several months) before the simultaneous appearance of sensations of heaviness and pain in both lower limbs with the impossibility of distinguishing the predominant sensation. The patient’s medical history began in 2008 reporting pain and heaviness in the lower limbs with a debilitating tendency, prompting a consultation with a neurologist followed by that with an infectious disease specialist due to the HTLV-1/2 positivity found by ELISA, in the plasma and cerebrospinal fluid of the patient. No other HIV-1/2, HCV, HBV serology was positive. Due to the lack of knowledge about treatments linked to HTLV-1, the infectious disease specialist referred the patient to a hematologist, where an immunosuppressant (imurel) was prescribed for one year. And a biopsy of the salivary glands was carried out following complaints of persistence of dry mouth. The results of the said biopsy highlighted a histological appearance compatible with chronic salivates, which could be sicca syndrome or that of Mikulicz-SjÖgren (grade 2). The evolution of the pathology under treatment was not satisfactory, which forced the patient to meet another physician in 2009, when a MRI of the lumbar region, a muscle biopsy and an autoimmune assessment were performed, and the results were in favor of polymyositis, despite the search for anti-nuclear antibodies was negative. Despite various treatments administered (corticosteroids, anti-inflammatories, immunosuppressants, minor chemotherapies) and associated simultaneously with physiotherapy, the patient’s condition improved only for a short period, from a few days to a couple of weeks, before the reappearance of symptoms with greater intensity than previously. In 2022, another MRI of the cervical, thoracic and lumbar region were carried out but once again, could not have been contributory, while the search for anti-nuclear antibodies was carried out in the same year and was positive. After a new consultation and examination by a neurologist in Morocco that the diagnosis of spastic paraparesis was established that same year. Table 6 below presents the different clinical and biological characteristics of inflammatory myopathies found in HTLV-1 positive patients.

**Table 6:** Clinical and biological comparison of HTLV-1 positives and negative myopathies.

Patient n°	19	100	218	79	328
Age^*^/Female	34/F	40/F	29/F	47/F	24/F
Creatine Phosphokinase activity (CPK)	N	≈1.1N	N		> 66N
Protein C reactive	> 112N	N	N		
Antinuclear antibodies	–	**+**	**+**		
**Muscular biopsy result**					
Inflammatory infiltrates (biopsy)	+	+	**+**		**+**
Necrosis (biopsy)					**+**
Neurogenic atrophy (biopsy)		**+**			**–**
Proximal muscular weakness	**+**	+	+	+	+
Myalgia	**+**	+	+	+	+
Atrophy d/p	+d/p	+d/p		–	+p
Cutaneous lesions	–	–	–	+	+
Hyper-reflexia (lower limbs)	+	–	+	–	–
Areflexia	–	+	–	–	–
Bladder dysfunction	+	+	+	–	–
Sensory disturbance	–	–	+	–	–
HTLV-1 result	+	+	+	–	–
Diagnosis	PM	PM	PM	DM	DM

N: Normal.

* Onset age of disease.

d/p: distal/proximal

PM: polymyositis

DM: dermatomyositis

### Assessing risk-factors for HTLV-1 infection in the studied population

Female genre was significantly at high risk of being HTLV-1 infected comparatively to male counterpart (AOR 4.75; 95% CI 1.60 - 14.08, Table 7). Similarly, we found a significant degree of association between the HTLV-1 infection and the birthplace with higher recorded prevalence in the South, Southeast and western of Gabon (*p-value* = 0.01). Having an asymptomatic/parent status corresponded to a low risk of being infected by HTLV-1 (AOR 0.84 95% CI 0.28 - 2.53). Also, no significant degree of association between HTLV-1 infection and other potential risk factors (socio-economic classes, service, age, etc...) was established. Although they were not significant, important HTLV-1 prevalence rates were observed with certain risk factors such as having been blood-transfused at least once in the lifetime (10.7% against 6.2%) and/or having experienced butchering activities of non-human primate (8.7% against 6%). Also, co-infection and comorbidity stood out as controversial factors, while HTLV-1 infectiousness seemed to be low among HIV-1 seronegative patients than those confirmed HIV-1 seropositive (AOR 0.47 95% CI 0.12-1.84). The HTLV-1 infection risk was highest in non-diabetic individuals, relative to those with a diabetic clinical status (AOR 1.3 95% CI 0.34-4.69). The HTLV-1 seems to go well with certain conditions and not with others, so signaling pathways involved in HTLV-pathogenesis need to be thoroughly investigated to determine the critical factors of such discrepancy.

**Table 7:** Assessing HTLV-1 risk factors and comorbidities in our study population.

	Category	N	Serological/Molecular Screening			
**Variable**	**n.positive**	**%**	**AOR (95% CI)**	***p-value***
**Gender** (Gender ratio M/F = 0.8)	Men	155	4	2.6	4.75 (1.60 - 14.08)	0.002
	Women	197	22	11.2		
**Age**	[0-14]	6	0	0	–	0.7
	[15-30]	54	4	7.4		
	[31-46]	88	4	4.5		
	[47-62]	125	10	8		
	≥63	79	8	10.1		
**Status**	Parent	62	4	6.4	0.84 (0.28 - 2.53)	0.9
	Patient	290	22	7.6		
**Socio-economic class**	Students	44	2	4.5	–	0.5
	Low-skilled workers	179	17	9.5		
	Skilled workers	92	6	6.5		
**Service**	Dermatology	11	2	18.2		0.7
	Internal Medicine	152	13	8.6		
	Neurology	189	11	5.8		
**Nationality**	Other citizens	26	0	0	–	0.2
	Gabonese	311	26	8.4		
**Birthplace**	Central Africa ^**^	8	0	0	–	0.01
	West Africa	18	0	0		
	Europe^*^	1	0	0		
	Estuaire	97	3	3.1		
	Haut-Ogooué	36	4	11.1		
	Moyen-Ogooué	17	2	11.8		
	Ngounié	41	7	17.1		
	Nyanga	24	0	0		
	Ogooué-Ivindo	10	0	0		
	Ogooué-Lolo	17	4	23.5		
	Ogooué maritime	22	4	18.2		
	Woleu-Ntem	46	2	4.3		
**Blood transfusion history**	Yes	103	11	10.7	–	0.2
	No	242	15	6.2		
	Unknown	7	0	0		
**NHP butchering history**	Yes	173	15	8.7	–	0.6
	No	167	10	6		
	Unknown	9	1	11.1		
**Diabetes**	Yes	41	3	7.3	1.3 (0.34 - 4.69)	0.9
	No	133	12	9		
**HIV**	Yes	21	3	14.3	0.47 (0.12 - 1.84)	0.4
	No	164	12	7.3		
**Breastfeeding**	Yes	331	26	7.8	–	1
	No	6	0	0		

N: total size

n + : number of positive

%: percentage of positive

Nb: number

NHP: non-human primate

AOR: Adjusted Odd Ratio determined only where applicable

* Not included in the test because of insufficient data

** Except Gabon

### Phylogenetic analysis

**Fig 1:**
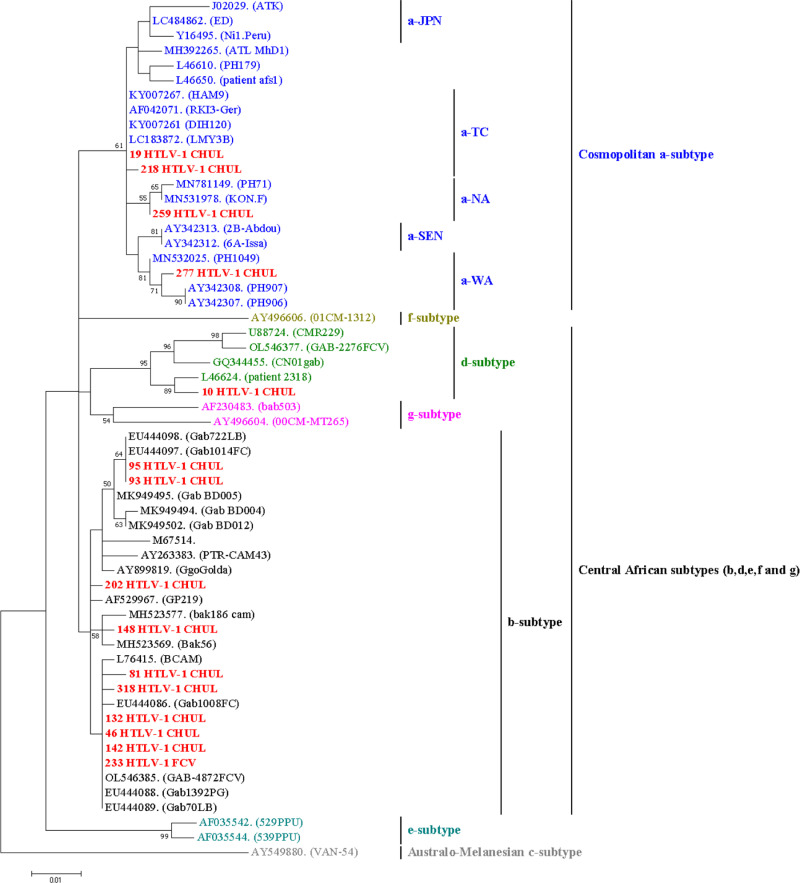
Phylogenetic tree for HTLV-1 Env gene. The tree was generated by the Maximum Likelihood method with GTR model from fragments of 522-pb, encoding for the end of the carboxyl terminus of the product of the gp46 gene and most of the product of the gp21 gene. All were obtained from 58 HTLV-1 isolates, including 15 new strains from our study (in red) and 43 other strains that were previously published in the GenBank. The accession numbers of the new sequences from patients are PQ367264-PQ367278. The tree was replicated 1000 times and horizontal branch lengths are drawn to scale, with the bar indicating 0.01 nucleotide replacements per site.

Phylogenetic analysis was finally carried out on 15 HTLV-1 strains among the 19 amplified in the 522-bp segment of the env region. Blast research and phylogenic analyses showed that 10 of the new 15 characterized strains belonged to the predominant Central African genotype HTLV-1b, while the other 4 belonged to the cosmopolitan subtype HTLV-1a and mainly to the subclades a-TC, a-Na and a-Wa (Fig 1). The remaining one strain belonged to the rare subtype from Central African HTLV-1d. Phylogenic analyses also reported a high molecular diversity among HTLV-1b genotype, characterized by the existence of several subclades. According to the assessment of nucleotide divergence of HTLV-1 strains isolated genotypes, all HTLV-1b strains were closely related with a slightly nucleotide divergence of 0.3 to 1.6%. There were 8 unique sequences that differed by 2–8 nucleotides and two positive-patients were found with the same strain of HTLV-1b (93_HTLV-1_CHUL and 95_HTLV-1_CHUL). Concerning the four HTLV-1a genotypes isolated in this work, they display a nucleotide divergence of 1.2 to 1.6% between them and 1.5 to 2.6% divergence to the HTLV-1 reference strain ATK from Japan.

### HTLV-1 intrafamilial transmission

**Fig 2:**
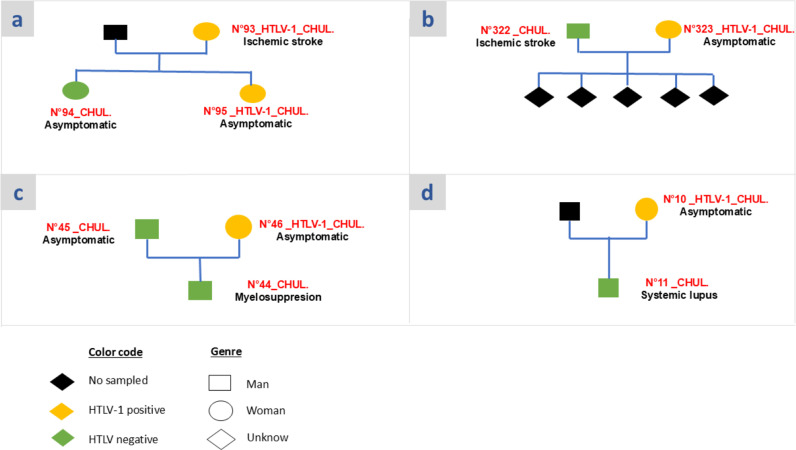
Pedigree of the four families with patients’ family members infected with HTLV-1 in this work. The identities (ID) of participants are represented in red while just below the ID, the corresponding disease is mentioned. All cases of asymptomatic individuals referring to a patient’s family member as mentioned above.

As previously mentioned, only 4 of the 62 patients’ relatives or family members sampled were positive for HTLV-1 infection after all serological and molecular tests (Table 7). Among these four HTLV-1 positive individuals, only 1 (n° 95 HTLV-1_CHUL, a 56-year-old female teacher) had a mother (n°93 HTLV-1_CHUL with 70-year-old) who suffered from an ischemic stroke and was also positive for HTLV-1 (Fig 2A below). Phylogenetic and Blast analyses reported that these HTLV-1 strains isolated from these two related-women were close and identical; a 100% nucleotide identity on the Env gene analyzed fragment. The epidemiological data recorded during the daughter’s sampling time reported that she was never blood-transfused but breastfed for a longtime after delivery (over 6 months), suggesting a HTLV-1 mother-child transmission by breast feeding.

## Discussion

According to previous data, diseases associated with HTLV-1 are rare and rapidly fatal in some cases, which still constitute real challenges in assessing the prevalence of induced pathologies or/and impact on public health [8,33]. This study is one of the first clinical studies that attempted to assess the prevalence of HTLV-1 associated diseases in Gabon, as well as the first that clearly highlights the situation of HTLV-1 associated diseases, which are underdiagnosed. Also, we report here 7.39% (26/352) overall prevalence rate of HTLV-1, among individuals attending the main teaching hospital in Gabon, which is similar to the general prevalence of HTLV-1 (7.3 to 8.7%), as previously reported in epidemiological studies conducted in rural adult population of Gabon [6,17]. Moreover, we report a high prevalence of diseases associated with HTLV-1, estimated at 3.7% (10/272), which was recorded among the diagnosed diseases for a total number of 265 patients. Although, no significant association between HTLV-1 infection and the observed diseases (*p-value* = 0.07, Table 3) was found, this could be due to both the diversity of diagnosed diseases recorded and the small number of patients sampled for each HTLV-1 associated disease.

We report 55.6% (5/9) of HTLV-1 positivity among patients with myelopathies, which is quite different to the 96% (25/26) and 1.6%-15% (for 1/61 and 3/20) reported among patients with spastic paraparesis in Zaïre (actual Democratic Republic of Congo, DRC) and Ivory Coast, respectively [34–36]. The difference with the prevalence rates from Zaïre and the Ivory Coast could be explained, on the one hand, by the lower number of patient cases in our study, and on the other hand, by the patient selection criteria or the diagnosis chosen. Also, due to the diversity of diagnoses recorded in our study, we considered patients with similar diagnoses. Thus, in our study, 2 out of 9 patients were with a motor deficit of the lower limbs and did not present any typical symptoms of TSP, whereas in those studies from Zaire and Ivory Coast, the population studied was in most cases made up of only individuals or patients with typical TSP [34–36]. All our 5 patients with TSP were women, with a mean age of onset disease of 40.8 ± 17.6 years (for 4/5 of them), consistent with previously reported data. Indeed, it is known that TSP/HAM occurs mainly in adult women, whose average age of onset is often between 40 to 50 years compared to men [37,38]. However, the reported TSP/HAM prevalence rates in this study were similar to 59% (10/17) and 66% (24/36) reported in Martinique and black-Zulu South African patients, respectively [10,37]. The clinical characteristics of TSP/HAM that we report here for the 5 patients seem to agree with those already described elsewhere in other endemic countries including Zaïre, South Africa, Peru, Brazil, Japan, etc. [30,34,37,39]. In other words, most of our TSP-positive patients (3/4) presented a long-term course of the disease (15.7 years ± 6) with bladder and sphincter problems, coupled with low back pain in 4/4 patients. Proximal weakness of the lower limbs was noted in all 4 patients, while hyperreflexia of the lower limbs was found in the 5 positive patients with spastic paraparesis. Only three patients were examined for lower limb sensory disturbances, and two (2/3) were with the symptom. Among our 5 HTLV-1 positive patients with TSP/HAM, only two had an insidious onset (Table 4). Thus, the remaining 3 patients reported a sudden onset, mainly characterized by a sensation of electric shock (2/3, no. 218 and no. 233) and acute pain in the lower limbs (1/3, no. 277). That sudden onset disease, although relatively rare, is sometimes reported with the onset of TSP/HAM [30,40]. Neurological physicians had agreed to a spastic paraparesis as diagnosis in those 5 HTLV-1 positive patients, but only one (1/5) of them was previously tested in 2007 for HTLV-1/2 by ELISA, for screening antibodies in sera and the cerebrospinal fluid (CSF). Although, useful as diagnostic guidance tests for HTLV, ELISA tests alone remain insufficient for confirmation of any given infection, as false positives might occur. For the 4 remaining patients, their HTLV-1 positivity was confirmed by ELISA, WB and PCR were performed during this study, with two (n°19 and n°277, Table 4) of them, who were frequently seen by physicians for their conditions.

In addition to TSP/HAM, we also report 10.5% (2/19) of HTLV-1 prevalence among non-Hodgkin lymphomas (NHL), 2 suspected ATL cases with an acute and lymphomatous forms. The prevalence of ATL that we describe in this study seems slightly lower than the 15.4% (4/26) prevalence of ATL reported by Delaporte and co-workers in the same hospital in 1993 [20]. One potential explanation for this difference could be the larger number of participants, which was approximately 1.4 more than that of this current study. Similarly, we also report a lower prevalence of ATL than that of 21.4% (3/14) previously reported in Bamako (Mali) in 1998, among patients with cutaneous lymphomas [41]. The average age of 58-years of their patients was older than 44- years reported in this study as well as their lower number of patients could explain the difference of prevalence rates. Some cases of ATL have already been described in many African countries, and most of those appear to be primarily case reports [13]. Among our two HTLV-1 positive patients with non-Hodgkin’s lymphoma, just 1/2 was previously tested for HTLV-1/2 antibodies screening. The ATL diagnosis is mainly based on the proof of mature T CD4 + monoclonal proliferation, all containing the HTLV-1 provirus at the same integration site [42,43]. Unfortunately, for both patients, no flow cytometry analysis was performed, which could have given a better understanding. Furthermore, the histopathological analysis of lymph nodes was just performed for one patient and was not substantial. Also, no immunohistochemistry or southern blot analysis were performed for these patients, which really constitutes a limit in this diagnosis. In fact, as the HTLV-1 infection is known to be mostly common in low-income social economic class people [6,17], a healthcare coverage as this of Gabon, which faces many challenges and could strongly hinder the ATL diagnosis for many patients. Additionally, the high cost of examinations and shortage of diagnostic materials in many laboratories, as well as the lack of awareness of medical staff about ATL disease, all could be reasons that explain the underdiagnoses of ATL cases and the low reporting of cases until nowadays in Gabon.

Beyond TSP/HAM and the two suspected ATL cases, to our knowledge, this study is probably the first to describe some cases of polymyositis potentially induced by HTLV-1 in Gabon. The HTLV-1-associated polymyositis is considered a rare disease [44]. The HTLV-1-associated inflammatory myopathies have already been reported with high prevalence rates in most cases in patients elsewhere including those from West Africa, West Indies, Jamaica, Martinique, Japan and other endemic areas [44–47]. Until nowadays, this was not yet the case in Gabon [18]. Here, we predominantly report HTLV-1 positivity in patients (3/5 or 60%) with inflammatory myopathies, which represents a prevalence approximately 7 to 8 folder higher than HTLV-1 prevalence reported in the rural adult population in Gabon [6,17]. This prevalence seems consistent with 63% (24/38) and 50% (7/14) reported in Jamaica and Martinique, respectively [11,44]. Nevertheless, it seems higher than the 27.5% (11/40) recorded in Kagoshima, Japan [48]. Once again, the difference between our prevalence and that of Kagoshima could result from the small size of the studied population or number of patients. The mechanisms of induction of HTLV-1 polymyositis remain poorly understood. A direct infection of skeletal muscle fibers by HTLV-1 was described in a case of polymyositis with co-infection by HTLV-1 and HIV-1, but this study was quickly contradicted by the evidence of the real integration of the HTLV-1 provirus in internal mononuclear inflammatory cells (mainly CD4+ T cells) that have infiltrated skeletal muscle [49,50]. In all cases, an attack on the immune system characterized by a lack of self-recognition and a high production of HTLV-1 induced inflammatory cytokines by means of the Tax protein could explain the pathophysiology of this disease [51]. Although, a direct link between HTLV-1 infection and myopathies has not yet been established, in the HTLV-1 endemic area, the high prevalence of HTLV-1 reported in patients with this pathology is higher than that of the general population and blood donors in most of the cases [11,45,48]. That could suggest a potential link between this viral infection and this disease. In addition, numerous studies have reported cases of polymyositis patients infected with HTLV-1, who also developed HTLV-1-associated neurological symptoms including TSP/HAM [48,52,53]. Indeed, in this study, two of three polymyositis cases (2/3) developed TSP/HAM after many years of illness (No. 19 and No. 218) as seen in this study (Table 4). Thus, given the possibility that HTLV-1 infection also induces polymyositis, one of the main remaining problems may be differentiating factors between the idiopathic form of polymyositis and that caused by HTLV-1. Currently, it does not yet seem to be any precise criteria for discriminating between these two forms [11].

A case control study led by Desdouits and co-workers in 13 patients with HTLV-1-associated inflammatory myopathies (HAIM) from West Africa and West India reported some histological alterations and inflammation in patients’ muscles, with classical features of idiopathic myositis [46]. Furthermore, a high anti-HTLV-1 antibodies titers with low proviral load was obtained and compared to that observed among asymptomatic HTLV-1 carriers, who were also noticed among patients with HAIM [46].

Also, some similar studies should be conducted in Gabon to assess the histological and virological features of HAIM to better discriminate idiopathic polymyositis and those induced by HTLV-1.

In the current study, we observed that HTLV-1 infection is seemingly associated with the gender, as women are mostly infected and affected than men, and that the prevalence is directly proportional to the age; the prevalence increases with age and higher prevalence rates were observed among older people as seen (Table 7). These findings corroborate with previous data and are already known as characteristics of HTLV-1 epidemiology [2,6,17].

We also report a significant association between HTLV-1 infection and the birthplace (*p-value* = 0.01) with high prevalence recorded among people from Ogooué-Lolo (23.5%, 4/17), a province that is seemingly a rural region. This province was previously described as one of the most endemic provinces for HTLV-1 infection in Gabon [6,17]. The link between HTLV-1 infection and birthplace in Gabon could be explained by socio-cultural behaviors such as the practice of initiation rites with scarifications, marriage between people living in the same endemic-region or from the same ethnical group. However, HTLV-1 acquisition was also previously associated with multiple hospitalizations among rural adult population, suggesting that HTLV-1 potentially involves nosocomial transmission in some rural regions in Gabon [6].

No association between HTLV-1 infection and NHP butchering activities was found although HTLV-1 prevalence was higher among people practicing this type of activity than those without. Also, the HTLV-1 infection was not related to blood transfusion as previously described, despite its prevalence was higher among people with this type of antecedent than those without (10.7% versus 6.2%). Association between HTLV-1 infection and blood-transfusion was much reported in Gabon and elsewhere, both in great epidemiological studies [6,17,54,55]. So, this type of result could be due to the low number of patients samples included in this work.

On the molecular level, most of characterized new HTLV-1 strains (10/15) belonged to the Central African genotype HTLV-1b, the main circulating in Gabon [6,7,13,17,28]. We isolated one HTLV-1 genotype d, which is occasionally reported in Gabon [17,23,56]. Four of HTLV-1 characterized strains belonged to the cosmopolitan HTLV-1a subtype that is known to be very scarce in Gabon [28]. Thus, this subtype was isolated from two Gabonese women; the first infected by the HTLV-1a subtype a-Na and the second by the HTLV-1a subclade a-Wa, both prevalent in North and West Africa, respectively [13,23,57]. Furthermore, we also reported among Gabonese patients, two other strains in women; the circulation of a-TC subclade from HTLV-1a genotype, which is the most disseminated subclade throughout the world [2,13,57].

Previously, Etenna and co-workers reported in 2008 a HTLV-1 prevalence of 2.1% (19/907) among pregnant women from 5/9 of provinces of Gabon [28]. Earlier in 1986, a prospective study based on follow-up of 4-years old babies born from HTLV-1 infected mothers reported a prevalence of HTLV-1 transmission to 15% (5/34), and estimated the risk of seroconversion to anti-HTLV-1 antibodies to 17,5% in the third most endemic province of Gabon for HTLV-1 infection [3]. In the current work, we reported one case of HTLV-1 mother to child transmission, which correlated with previous studies and pointed out this route of transmission of HTLV-1 as still relevant and probably a major player in the dissemination of this retrovirus in this county.

In summary, we report a high prevalence of HTLV-1 associated diseases in Gabon as well as the existence of HTLV-1 intrafamilial transmissions. These findings should attract the attention of government authorities, to improve the healthcare system by implementation of systematic screening for HTLV-1 in the groups of blood donors and pregnant women. However, popularizing the existence of HTLV-1 through awareness campaigns could also help in better understanding and fighting this infection, associated challenges and danger, as well as how to take necessary precautions.

## Supporting information

S1 Fig Number of patients affected per condition according to the clinical diagnosis. (TIF)

S2 Fig Some HTLV-1 positive patients with spastic paraparesis. N°277 (a and b) chronic spastic paraparesis with 15 years old evolution time in disease and presenting both low limbs muscular atrophies (b) daily requiring using of wheelchair. S2C Fig shows patient N°259 with spastic paraparesis who was daily on bed and presented cognitive impairment and dementia while the S2D Fig, a nurse patient (N°218) with chronic spastic paraparesis with 22 years old evolution time and requiring walking with bilateral supports.
(TIFF)

S3 Fig Cervical lymph node and floral T cell observed in peripheral blood tissues of patient. (N° 148) with acute form of ATL.
(TIF)

S4 Fig Tetraparesis in HTLV-1 positive patient. Patient suffering of polymyositis with 8 years of evolution and amyotrophy of forearms (S4B Fig).
(TIF)
