# Supplementary figures and images for: Clinical and molecular evidences of HTLV-1 infection in inpatients diagnosed with diseases previously described as associated to this infection: A case series in Gabon, Central Africa
Source: PLoS Negl Trop Dis. 2025 May 14;19(5):e0013075. doi: 10.1371/journal.pntd.0013075 (PMC12101779; doi:10.1371/journal.pntd.0013075)

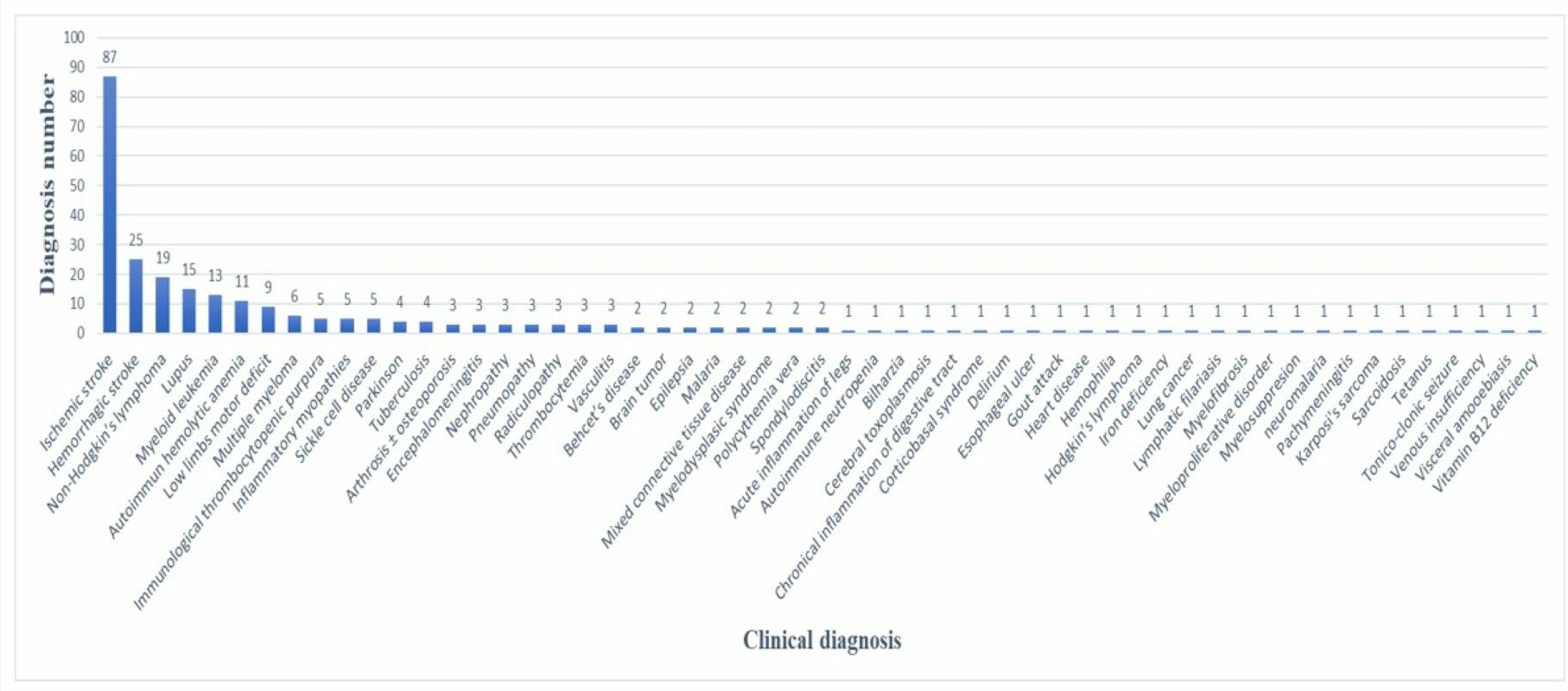

Supplement: S1 Fig — (TIF) [file pntd.0013075.s001.tif]

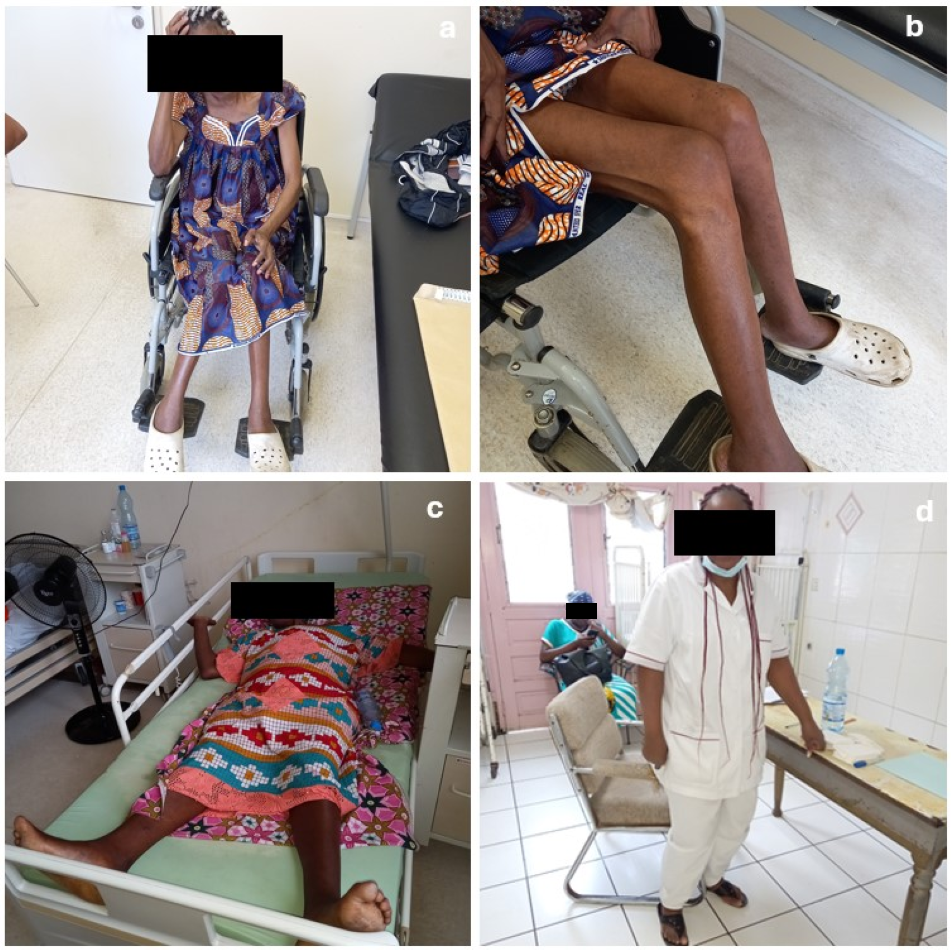

Supplement: S2 Fig — N°277 (a and b) chronic spastic paraparesis with 15 years old evolution time in disease and presenting both low limbs muscular atrophies (b) daily requiring using of wheelchair. S2C Fig shows patient N°259 with spastic paraparesis who was daily on bed and presented cognitive impairment and dementia while the S2D Fig, a nurse patient (N°218) with chronic spastic paraparesis with 22 years old evolution time and requiring walking with bilateral supports. (TIFF) [file pntd.0013075.s002.tiff]

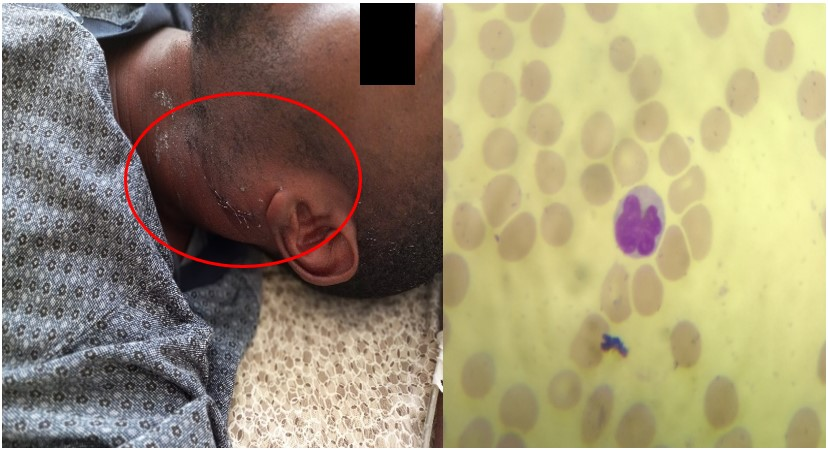

Supplement: S3 Fig — (N° 148) with acute form of ATL. (TIF) [file pntd.0013075.s003.tif]

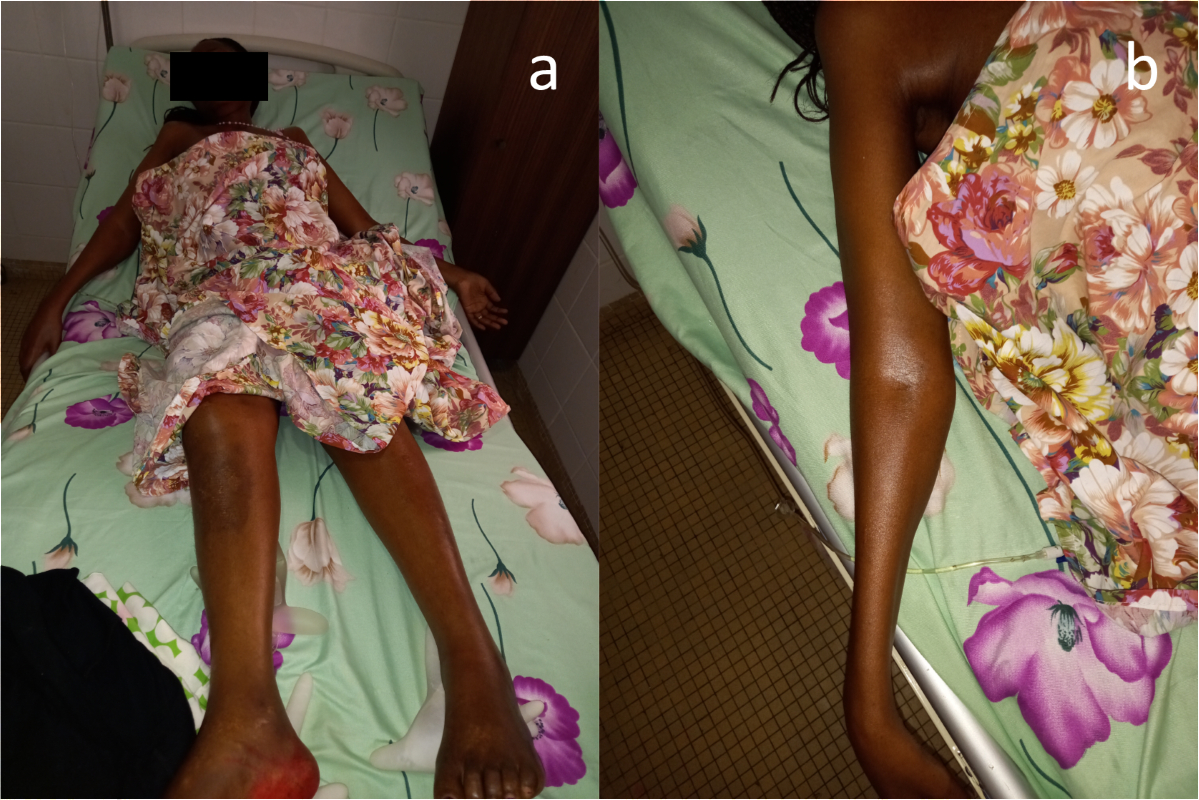

Supplement: S4 Fig — Patient suffering of polymyositis with 8 years of evolution and amyotrophy of forearms (S4B Fig). (TIF) [file pntd.0013075.s004.tif]
